# Clinical value of selected markers of angiogenesis, inflammation, insulin resistance and obesity in type 1 endometrial cancer

**DOI:** 10.1186/s12885-020-07415-x

**Published:** 2020-09-25

**Authors:** Katarzyna M. Terlikowska, Bozena Dobrzycka, Robert Terlikowski, Anna Sienkiewicz, Maciej Kinalski, Slawomir J. Terlikowski

**Affiliations:** 1 Department of Food Biotechnology, Medical University of Bialystok, Szpitalna 37 Street, 15-295 Bialystok, Poland; 2 Department of Gynecology and Obstetrics, Medical University of Bialystok, M. Sklodowskiej-Curie 24A Street, 15-089 Bialystok, Poland; 3 Department of Rehabilitation, Medical University of Bialystok, M. Sklodowskiej-Curie 24A Street, 15-089 Bialystok, Poland; 4 Department of Gynecology and Obstetrics of the Independent Public Healthcare Facility Regional Complex Jan Sniadecki Hospital, M. Sklodowskiej-Curie 26 Street, 15-950 Bialystok, Poland; 5 Department of Obstetrics, Gynaecology and Maternity Care, Medical University of Bialystok, Szpitalna 37 Street, 15-295 Bialystok, Poland

**Keywords:** Obesity, Insulin resistance, Angiogenesis, Inflammation, Endometrial cancer

## Abstract

**Background:**

It is a well-known fact show that the risk of developing endometrial cancer (type 1 EC) is strongly associated with obesity. In this study, selected markers, such as obesity, insulin resistance, angiogenesis and inflammation markers related to EC type 1 progression and patients’ survival data were analyzed.

**Methods:**

To measure levels of adiponectin, C-reactive protein (CRP), vascular endothelial growth factor-A (VEGF-A), angiopoietin-2 (Ang-2), insulin-like growth factor-1 (IGF-1), insulin and C-peptide in 176 preoperative serum samples, the immunoassay technique (EMIT) has been applied.

**Results:**

Angiopoietin-2 levels increase with age (*P* = 0.005), FIGO stage (*p* = 0.042), myometrial invasion (*P* = 0.009) and LVSI (*P* < 0.001). The CRP levels increase with age (*P* = 0.01), as well as the advancement of the FIGO stage (*P* < 0.001), higher tumor grade (*P* = 0.012), and myometrial invasion (*P* < 0.001). A positive correlation between serum Ang-2 and CRP levels was demonstrated (*r* = 0.44; *p* < 0.001). Kaplan-Meier survival analysis showed that patients with high CRP levels in serum and Ang-2 presented a worse outcome (*P* = 0.03 and *P* = 0.015, respectively). Cox regression analysis of individual predictors revealed that high serum levels of Ang-2, CRP, advanced clinical FIGO stage (*P* < 0.001, respectively), old age (*P* = 0.013) were all significant overall survival predictors. By means of multivariate analysis, their predictive significance was confirmed.

**Conclusion:**

Our study provides evidence that serum levels of Ang-2 and CRP may serve as predictors for assessment of the clinical stage of type 1 EC and are significantly associated with poor prognosis. It is likely that angiogenesis and inflammation associated with obesity have a significant impact on EC type 1 progression and survival rate of patients.

## Background

Endometrial cancer (EC) is one of the most common gynecological malignancy. According to 2018 Global cancer statistics 382,069 women have been diagnosed with endometrial cancer worldwide [1]. In Poland, the *age-adjusted incidence* rate was 16,61 per 100,000 women in 2018 with 6243 new incidents and 1690 deaths [2].

The dualistic model for EC divides this malignancy into two main categories: type 1 estrogen-dependent endometrioid carcinoma (G1 and G2) and type 2 non-endometrioid carcinoma (G3). Type 1 tumors account for about 80–85% of all endometrial malignancies and are most often linked to a good clinical outcome. Not only the FIGO stage but also tumor grade, depth of myometrial invasion, LVSI and lymph node status are the most valuable clinicopathological prognostic variables [3, 4].

Obesity is correlated with a chronic, low-grade inflammation which is characterized by raised systemic levels of inflammatory markers which also correlate obesity with the risk of EC [5–9]. Increased adipose tissue mass may be contributing to the development of cancer by elevated production of pro-inflammatory cytokines and chemokines. Several studies confirmed that CRP may influence the secretion of inflammatory cytokines, thus increase the risk of EC [9–11]. Immune cells that secrete the proinflammatory cytokines, such as tumor necrosis factor α (TNF-α) and interleukin-6 (IL-6) can infiltrate adipose tissue. IL-6 triggers the production of CRP in the liver - this acute phase protein is well-known for its pro-inflammatory properties [11]. Circulating adipokines, like adiponectin, have a systemic immunomodulatory effect, also play an important role in the development of selected types of cancer. Prospective studies have shown that insulin, IGFBP2, leptin, adiponectin, and C-peptide play an important role in the development of EC [12]. Most cancer-related epidemiological studies have revealed that the risk for type 1 EC development is strongly correlated with obesity. Such correlations have been noticed in both pre- and postmenopausal women as well as in cohort and case-controlled trials. Postmenopausal obesity refers to increased circulating estrogens which are attributable to the aromatization of androgens in adipose tissue [13]. Obesity is found to be associated with lower levels of sex hormone-binding globulin (SHBG), which leads both, to higher bioavailable levels of estrogen and higher insulin levels. That, in consequence, may increase the risk of EC development [9]. The association of EC and diabetes have been shown in three meta-analyses [14–16]. Several factors including insulin resistance, higher leptin levels, lower adiponectin levels, and chronic inflammation are considered to be important factors in obesity-related carcinogenesis.

Neoangiogenesis sustains the growth, advancement, and metastasis of solid tumors. Vascular endothelial growth factor (VEGF) and angiopoietin 2 (Ang-2) are important regulators of neoangiogenesis in the endometrium [17]. Angiopoietin-2, Tie2 ligand-receptor, and VEGF-mediated pathway are found to be crucial for the regulation of vascular maturation or stability, having been implicated in the control of physiological angiogenesis [18, 19].

When the primary tumor volume grows, intratumoral hypoxia increases Ang-2 expression to promote angiogenesis for tumor metastasis. Hypoxic cancer cells mediate functional interactions that promote angiogenesis, lymphangiogenesis, and metastasis [20]. In lung cancer patients it was shown that higher serum levels of Ang-2 were associated with a significantly poorer prognosis. Thus, Ang-2 and Ang-2 mRNA in tissue or sera are thought to be useful diagnostic biomarkers [19, 21].

In the present study, we examined a correlation of several markers related to obesity, insulin resistance, neoangiogenesis and inflammation with type 1 EC development and patients’ survival.

## Methods

### Patients

The current study was conducted on a cohort of 176 Caucasian women suffering from type 1 EC, treated in the Gynecology and Obstetrics Department of the Independent Public Healthcare Facility Regional Complex Jan Sniadecki Hospital in Bialystok, Poland in 2006–2012. The study protocol was approved by the Bioethics Committee at the Medical University of Bialystok, Poland (R-I-003/177/2004). Enrolled patients were informed of the study’s purpose and gave their consent for the study. All patients were treated surgically in accordance with FIGO criteria. The standard blood tests, chest radiographs, and abdominal ultrasound tests including pelvis were performed. In several cases, CT or MRI was performed too. Performed procedures included a hysterectomy with bilateral salpingo-oophorectomy (*n* = 83), hysterectomy with bilateral salpingo-oophorectomy, and bilateral pelvic/paraaortic lymphadenectomy (*n* = 93). Patients included in this study had not been given any preoperative treatment.

Histopathological examination was performed according to the WHO guidelines and classification. To confirm the clinical FIGO stage, the depth of myometrial invasion, histopathological tumor type and grade, and the absence or presence of LVSI and prepare for light microscopy examination, all representative tissue samples were H&E stained. To avoid any misinterpretations, the surgical specimens were presented to two gynecologic pathologists for an independent review.

### Collection and storage of samples

Blood samples were collected into a serum separator tube (Vacutainer, Becton-Dickinson, USA) and allowed blood to clot at room temperature for 30 min. All samples were centrifuged at 3000 g for 10 min then collected; the supernatant was stored at − 80 °C until examination.

### Immunoassays

We used commercially available Quantikine human ELISA kits (R&D systems, Minneapolis, MN, USA) for adiponectin, high sensitivity-C-reactive protein (CRP), VEGF-A, Ang-2 and IGF-1 and human ELISA kit (Millipore, Billerica, MA, USA) for insulin and C-peptide to measure protein levels in the patients’ serum samples. All ELISAs were carried out according to manufacturers’ instructions and samples were assayed in duplicate according to proper control standards. Human Total Adiponectin/Acrp30 Quantikine ELISA Kit DRP300 (sensitivity: 0.891 ng/ml, assay range: 3.9–250 ng/ml) Human C-Reactive Protein/CRP Quantikine ELISA Kit DCRP00 (sensitivity: 0.022 ng/ml, assay range: 0.8–50 ng/ml Human VEGF Quantikine ELISA Kit DVE00 (sensitivity: 9 pg/ml, assay range: 31.3–2000 pg/ml) Human Angiopoietin-2 Quantikine ELISA Kit DANG20 (sensitivity: 21.3 pg/ml, assay range: 46.9–3000 pg/ml) Human IGF-I Quantikine ELISA Kit DG100 (sensitivity: 0.056 ng/ml, assay range: 0.1–6 ng/mL Human Insulin ELISA EZHI-14 K Millipore (sensitivity: the lowest level of insulin that can be detected by this assay is 0.85 μU/mL when using a 20 μl sample size, specificity: 100%). Human C-Peptide ELISA EZHCP-20 K Millipore (sensitivity: the lowest level of Human C-Peptide that can be detected by this assay is 0.05 ng/ml, specificity: 100%). Personnel running the assays was not informed of patients’ clinical status, and the results were disclosed to the surgeons only after recording patients’ disease status. The test precision for markers was performed in accordance with the protocol guidelines of the Clinical and Laboratory Standards Institute (CLSI) [22].

### Data collection

Demographic and clinical data, as well as the pathology report for every patient, have been prospectively stored in the hospital database. Baseline height, weight, and BMI have been acquired from medical records along with the follow-up information. The BMI was established in consideration of the World Health Organization classification: < 18,5 kg/m^2^ - underweight; 18,5–24.9 kg/m^2^ - normal; 25–29.9 kg/m^2^ - overweight; ≥30 kg/m^2^ - obese [23]. The BMI decreased with age > 60 years. Only 1.1% of patients appeared to be underweight, 8.5% were normal, 52.3% - overweight and 38.1% were obese, among them 5.4% morbidly obese. Twenty-one patients (11.9%) were diagnosed with diabetes mellitus type 2. All follow-ups were concluded before 30 September 2018.

### Statistical analysis

Statistical analysis was performed in Statistica software package 13.3 PL (StatSoft, Inc. StatSoft Poland Ltd.). Frequency and descriptive statistics were applied to characterize the cohort. Independent-sample T-tests to compare serum markers levels in patients with or without LVSI, myometrial invasion, FIGO stage, grade, and age were used (or Mann-Whitney-U test when appropriate). Correlation between the selected markers of angiogenesis, inflammation, insulin resistance and obesity was assessed using Pearson’s correlation analysis. Biomarkers that showed significant correlation were analyzed by linear regression to determine the working relationships between the biomarkers. We successively conducted both Kaplan-Meier and Cox regression analyses so that we could analyze the overall survival of the patient. We applied medians in order to divide continuous data into groups for Kaplan-Meier analysis, with standard cut off points for BMI. For the continuous variables, hazard ratios were estimated using the following units: 100 units of VEGF-A, 1000 units of Ang-2, 1 unit of CRP, insulin, C-peptide, and BMI, 10 units of IGF-1 and per decade of age. Predictors were entered either on their own or jointly; stepwise procedures were not used. The Cox-proportional hazard model was used to assess the prognostic value of serums VEGF-A, Ang-2, Adiponectin, Insulin, C-peptide, CRP, IGF-1 and BMI as log-transformed continuous factors in univariate and Ang-2 and CRP in multivariate analyses. The base model consisted of traditional prognostic factors such as FIGO stage, age, tumor grade, myometrial invasion, and LVSI. Levels of VEGF-A, Ang-2, Adiponectin, Insulin, C-peptide, CRP, IGF-1 and BMI were entered separately in a second block. Points estimated were reported as hazard ratios (HRs) and 95% confidence intervals (CIs). *P* < 0.05 was found to be statistically significant.

## Results

The trial cohort consisted of 176 patients with type 1 EC. The clinical characteristics of study participants are summarised in Table 1. Patients ranged in age from 54 to 87 years (median = 69) with 67.6% of patients ≤60 and 32.4% > 60 years of age. All tumors were clinically, surgically and histopathologically categorized as follows: 131 patients (74.4%) were FIGO first stage, 23 (13.1%) - second stage, 18 (10.2%) - third stage and 4 (2.3%) - fourth stage (= stage IVA only). Lymphatic vascular space invasion (LVSI) was identified in 62 patients (35.2%). Myometrial invasion ≥50% was recorded in 97 (55.1%) out of 176 tumors. All samples were categorized according to their histological grade: 105 (59.7%) were found to be grade 1 and 71 (40.3%) were grade 2.

**Table 1:** Patients characteristics

	N	(%)
Total	176	(100)
Age (years)		
≤ 60	119	(67.6)
> 60	57	(32.4)
FIGO stage		
I	131	(74.4)
IA	79	(44.9)
IB	52	(29.5)
II	23	(13.1)
III	18	(10.2)
IV	4	(2.3)
Lymphatic vascular space invasion		
Yes	62	(35.2)
No	114	(64.8)
Myometrial invasion		
< 50%	79	(44.9)
≥ 50%	97	(55.1)
Histological grade		
G1	105	(59.7)
G2	71	(40.3)

Serum levels of the angiogenic factors VEGF-A and Ang-2 and the inflammatory factor CRP, with reference to clinicopathological features are displayed in Table 2. The VEGF-A levels were considerably elevated when LVSI and myometrial invasion (≥50%) was present (*P* = 0.013 and *P* = 0.002; respectively). Angiopoietin-2 levels increased with age (*P* = 0.005), myometrial invasion (*P* = 0.009), LVSI (*P* < 0.001) and FIGO stage (*P* = 0.042). The CRP levels increased with age (*P* = 0.01), FIGO stage (*P* < 0.001) and higher grade (*P* = 0.012), and also with myometrial invasion (*P* < 0.001).

**Table 2:** Serum angiogenic and inflammatory factors according to clinicopathological features in type 1 endometrial cancer patients

	N (%)	VEGF-A (pg/ml)	*P*-value	Ang-2 (pg/ml)	*P*-value	CRP (μg/ml)	*P*-value
		Mean (±SD)		Mean (±SD)		Mean (±SD)	
Total	176 (100)						
**Age (years)**							
≤ 60	119 (67.6)	414 (398)	0.632	2012 (891)	0.005	3.61 (2.68)	0.01
> 60	57 (32.4)	391 (370)		2948 (1246)		4.98 (3.29)	
**FIGO stage**							
I-II	154 (87.5)	347 (221)	0.061	2212 (1372)	0.042	3.46 (2.97)	< 0.001
III-IV	22 (12.5)	432 (443)		2968 (1314)		6.92 (3.17)	
**Lymphatic vascular space invasion**							
Yes	62 (35.2)	453 (312)	0.013	3102 (1652)	< 0.001	5.11 (3.09)	0.114
No	114 (64.8)	349 (305)		2451 (1232)		4.32 (3.39)	
**Myometrial invasion**							
< 50%	79 (44.9)	316 (181)	0.002	2183 (1386)	0.009	3.09 (2.97)	< 0.001
≥ 50%	97 (55.1)	411 (371)		2953 (1216)		5.11 (3.28)	
**Histological grade**							
G1	105 (59.7)	392 (402)	0.125	2562 (985)	0.135	2.54 (1.98)	0.012
G2	71 (40.3)	375 (328)		2746 (1134)		4.16 (2.97)	

Data for metabolic factors such as adiponectin, IGF-1, insulin, C-peptide and BMI values are presented in Table 3. Adiponectin levels increased with age (*P* = 0.001). There were no statistical differences found in the levels of IGF-1, insulin, C-peptide according to clinicopathological features.

**Table 3:** Obesity-related factors according to clinicopathological features in type 1 endometrial cancers patients

	N (%)	Adiponectin (ng/ml)	*P*-value	IGF-1 (ng/ml)	*P*-value	Insulin (μU/ml)	*P*-value	C-peptide (ng/ml)	*P*-value	BMI	*P*-value
		Mean (±SD)		Mean (±SD)		Mean (±SD)		Mean (±SD)		Mean (±SD)	
Total	176 (100)										
**Age (years)**											
≤ 60	119 (67.6)	6791 (4132)	0.001	98.41 (26.84)	0.082	18.43 (22.59)	0.132	4.19 (3.45)	0.392	29.42 (4.21)	0.001
> 60	57 (32.4)	8154 (5321)		95.35 (29.87)		14.98 (24.78)		4.93 (3.74)		27.14 (5.16)	
**FIGO stage**											
I-II	154 (87.5)	8632 (6244)	0.645	96.26 (35.84)	0.458	13.92 (17.92)	0.747	4.58 (3.64)	0.793	27.24 (5.26)	0.893
III-IV	22 (12.5)	8754 (5768)		87.82 (32.85)		14.12 (20.24)		4.78 (3.89)		27.92 (4.92)	
**Lymphatic vascular space invasion**											
Yes	62 (35.2)	8132 (5948)	0.821	91.21 (36.14)	0.452	16.82 (24.87)	0.604	4.73 (3.68)	0.894	27.42 (5.61)	0.793
No	114 64.8)	8241 (6005)		93.46 (33.86)		14.93(22.64)		4.81 (3.76)		27.29 (5.11)	
**Myometrial invasion**											
< 50%	79 (44.9)	8642 (6391)	0.729	94.18 (37.25)	0.864	12.56 (16.24)	0.151	4.45 (3.51)	0.934	27.56 (7.29)	0.391
≥ 50%	97 (55.1)	8294 (5943)		92.16 (34.91)		14.95 (24.62)		4.65 (3.14)		26.98 (4.73)	
**Histological grade**											
G1	105 59.7)	9134 (7424)	0.693	91.16 (27.23)	0.614	12.48 (14.68)	0.534	3.87 (2.32)	0.384	26.72 (5.72)	0.123
G2	71 (40.3)	9251 (5842)		97.37 (37.51)		15.96 (19.44)		4.62 (3.64)		26.98 (4.49)	

Pearson’s correlation analysis was performed to determine whether there were correlations between angiogenic, inflammation and obesity-related factors in type 1 endometrial cancer patients. There was a significant negative correlation between adiponectin and IGF-1, insulin, C–peptide and BMI (*r* = − 0.19; *p* < 0.001, *r* = − 017; *p* = 0.003, *r* = − 0.16; *p* = 0.02 and *r* = − 0.31; *p* < 0.001, respectively). A significant negative correlation was observed between CRP levels and IGF-1 (*r* = − 0.17; *p* < 0.001). The measured levels of VEGF-A were positively correlated with serum levels of Ang-2 and CRP (*r* = 0.18; *p* < 0.001, *r* = 0.25; *p* < 0.001, respectively). The Ang-2 levels showed a positive correlation with CRP and C-peptide (*r* = 0.44; *p* < 0.001, *r* = 0.16; *p* = 0.009, respectively). The C-peptide levels showed a positive correlation with IGF-1, insulin and BMI (*r* = 0.16; *p* = 0.01, *r* = 0.66; *p* < 0.001, *r* = 0.24; *p* < 0.001, respectively). A positive correlation was observed between IGF–1 and insulin (*r* = 0.17; *p* = 0.02) and insulin with BMI (*r* = 0.19; *p* < 0.001). No significant correlation was found between any other serum level combinations of analyzed markers (Table 4).

**Table 4:** Pearson’s correlation coefficient between angiogenic, inflammation and obesity-related factors in type 1 endometrial cancers patients

	VEGF-A	Ang-2	CRP	Adiponectin	IGF-1	Insulin	C-peptide	BMI
**VEGF-A**								
CC	1							
Significance								
**Ang-2**								
CC	0.18	1						
Significance	< 0.001							
**CRP**								
CC	0.25	0.44	1					
Significance	< 0.001	< 0.001						
**Adiponectin**								
CC	0.03	0.04	−0.03	1				
Significance	0.521	0.299	0.748					
**IGF-1**								
CC	0.12	−0.02	−0.17	−0.19	1			
Significance	0.071	0.834	< 0.001	< 0.001				
**Insulin**								
CC	0.04	0.03	−0.07	−0.17	0.17	1		
Significance	0.548	0.692	0.163	0.003	0.02			
**C-peptide**								
CC	0.03	0.16	−0.02	−0.16	0.16	0.66	1	
Significance	0.782	0.009	0.701	0.02	0.01	< 0.001		
**BMI**								
CC	0.03	−0.02	0.06	−0.31	0.08	0.19	0.24	1
Significance	0.421	0.538	0.292	< 0.001	0.116	< 0.001	< 0.001	

*CC* correlation coefficient

**Fig. 1:**
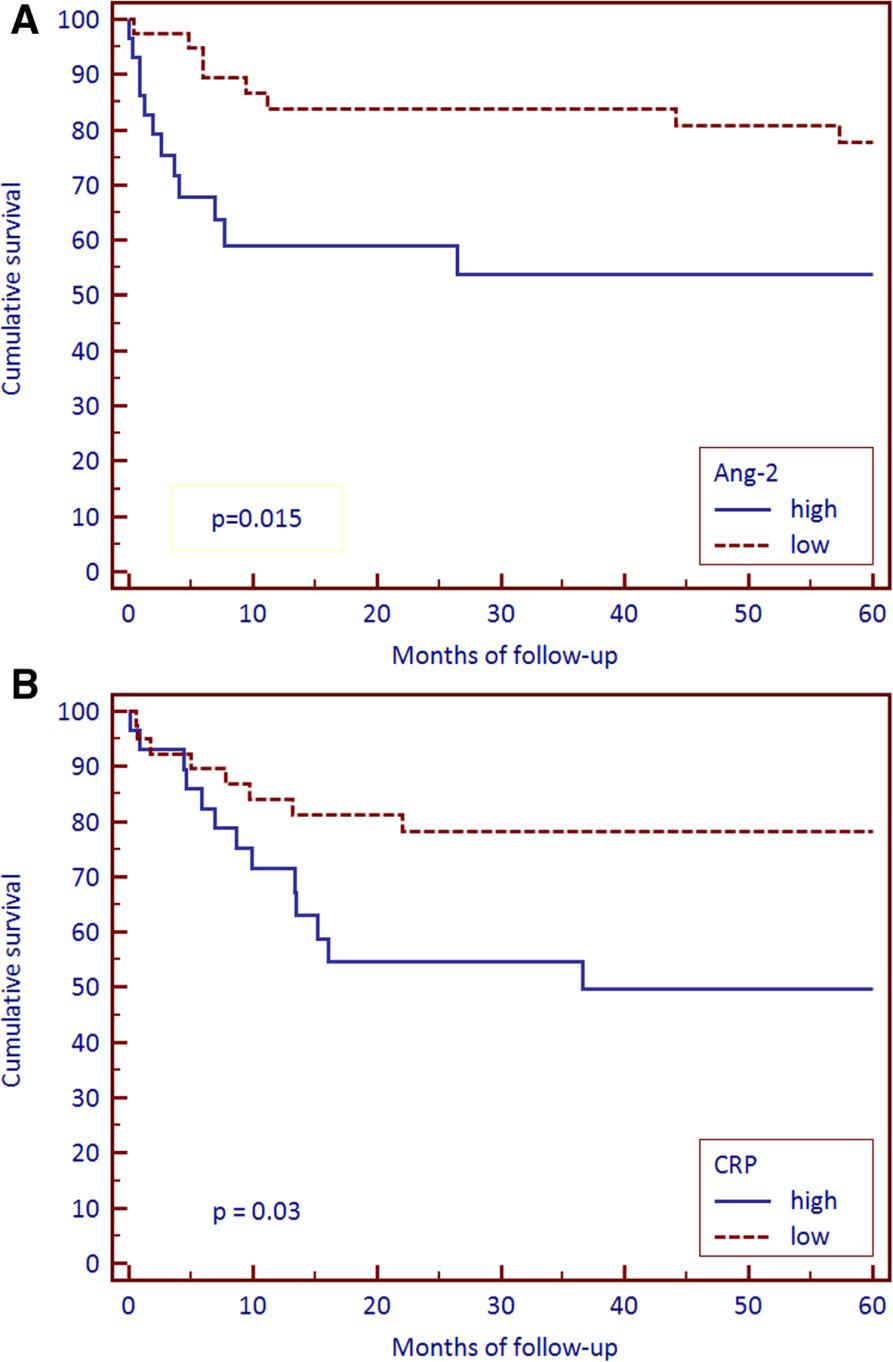
Survival of endometrioid endometrial cancer patients from surgery to death from any cause by Kaplan-Meier survival analysis. Survival between groups with high and low serum **a** Ang-2 and **b** CRP. Median values were used as cut-points for high vs low values

Within 5 years of observation, 44 patients died of various causes present in the cohort. Kaplan-Meier survival analysis revealed that patients with high serum levels of CRP (*P* = 0.03) and Ang-2 (*P* = 0.015) had significantly worse outcomes (Fig. 1a and b). Cox regression analysis of individual predictors revealed that Ang-2, CRP, FIGO stage (*P* < 0.001, respectively), LVSI (*P* = 0.009) and age (*P* = 0.013) appeared to be significant predictors of overall survival rate for the whole cohort. These predictors were then analyzed together in a multivariate analysis in which the predictive significance of Ang-2 (*P* = 0.006), CRP (*P* = 0.015), FIGO stage (*P* < 0.001) and age (*P* = 0.017) was confirmed (Table 5).

**Table 5:** Cox regression survival analyses

	Hazard ratio	95% CI for HR	Total *P*-value
**Univariate analysis**			
Base model			
FIGO stage			
III-IV vs I-II	5.98	3.11–9.89	< 0.001
Age (years)			
> 60 vs ≤60	2.31	1.24–4.58	0.013
Grade			
1 vs 2	2.59	1.16–4.86	0.062
Myometrial invasion			
> 50% vs ≤50%	3.28	1.83–5.92	< 0.001
LVSI			
yes vs no	2.63	1.24–4.76	0.009
Additions to model^a^			
VEGF-A	1.02	0.25–3.64	0.74
Ang-2	1.29	1.09–1.59	< 0.001
Adiponectin	1.01	0.99–1.06	0.923
Insulin	1.00	0.98–1.02	0.914
C-peptide	1.02	0.97–1.06	0.689
CRP	1.37	1.03–1.61	< 0.001
IGF-1	1.01	0.96–1.05	0.893
BMI	0.84	0.45–1.41	0.472
**Multivariate analysis**			
Base model			
FIGO stage			
III-IV vs I-II	3.57	1.79–6.94	< 0.001
Age (years)			
> 60 vs ≤60	2.21	1.19–4.32	0.017
Grade			
1 vs 2	1.52	1.18–2.96	0.683
Myometrial invasion			
> 50% vs ≤50%	1.11	0.49–2.09	0.861
LVSI			
yes vs no	1.16	0.53–2.36	0.734
Additions to model^b^			
Ang-2	1.31	1.02–1.69	0.006
CRP	1.22	1.01–1.43	0.015

^a^The base model consisted of traditional prognostic factors, and we separately entered the parameters in a second block

^b^Additions to the used model (all continuous, log-transformed, and separately entered)

## Discussion

The prevalence of EC is alarmingly high due to the increasing number of older women in populations and frequent cases of obesity. In fact, the 5 kg/m2 increase in BMI relates to a significant increase in EC [RR: 1.50 (1.42–1.59)] [24, 25]. Over 70% of women with type 1 EC are obese. For women with EC and BMI in the range 30–34.9, the RR of mortality was 2.53, and with BMI exceeding 40 the RR of mortality went up to 6.25. Women with BMI over 40 are characterized by significantly shorter survival rates and suffer from more endometrial cancer unrelated deaths when compared with non-obese women [8, 25–28]. Earlier studies suggest that BMI is negatively correlated with EC survival rate, although this association is questionable [29, 30]. In the current study, just like in the research by Mauland et al. [31] BMI had no independent prognostic impact on EC patients’ survival despite the fact that 90.4% of women with type 1 EC were overweight or obese. Our data demonstrate that women with type 1 EC enrolled in our study had their BMI associated with age and not with other clinicopathological factors.

Obesity contributes to the progression of metabolic syndrome, which can be identified by insulin resistance, which in turn is a risk factor for EC [25, 32, 33]. Chronic hyperinsulinemia causes the secretion of IGF-1 and lowers the production of IGF binding proteins, which consecutively heightens circulating levels of IGF. IGF-1 binding to the cognate receptor, IGF-1R, triggers a signaling cascade leading to proliferative and anti-apoptotic events [25, 34]. In our study pretreatment levels of adiponectin, IGF-1, C-peptide and insulin in blood did not correlate with clinicopathological variables and with patients’ cumulative survival. Similar results were obtained by Volkova et al. [35] in patients with colorectal cancer. Nevertheless, the individual contributions of these factors to obesity-related tumors, including type 1 EC, are not fully understood. However, it does not seem that any of the markers assessed here can be effectively used to define EC type 1.

Obesity changes profiles of cytokines and thus influences the chronic inflammatory conditions. Adipose tissue secretes leptin, VEGF, IL-1, IL-6, hepatocyte growth factor (HGF) and TNF-α that could induce tumor neoangiogenesis leading to the progression of solid tumors [25, 36]. Increased levels of angiogenic markers could be connected to poor outcomes and high grades in type 1 EC [25, 37]. In our previous study we have proved that preoperative serum VEGF levels could be a beneficial marker to foresee 5-year illness-free survival in type 2 EC [38].

It is a well-known fact that levels of Ang-2 are elevated in serum of adult patients with metabolic syndrome [39], as well as in some obese adults [40, 41], where Ang-2 is evidently correlated with vascular disorders [42]. A meta-analysis carried out by Xu et al. [21] showed that elevated levels of Ang-2 in lung cancer are associated with poor prognosis. Although the absolute participation of Ang-2 in cancer development is not evident so far, it is possible that higher levels of Ang-2, together with elevated levels of VEGF-A, have an additional effect on neoangiogenesis, as well as on the instability of new blood vessels [43]. Cullberg et al. [44] observed decrease of VEGF and Ang-2 levels in blood serum during weight loss that indicates their generally reduced angiogenic activity. In addition, these results indicate that VEGF-A and Ang-2 are associated with obesity and possibly with the development of other obesity-related diseases such as endometrial cancer. Volkova et al. [35] claim that serum Ang-2 levels were a stronger predictor of survival than serum VEGF-A levels, which are the main angiogenic factor associated with poor colorectal cancer outcomes [45]. In our study we found that in EC type 1 patients, serum levels of Ang-2 increased with the depth of myometrial infiltration, LVSI, and age. Especially patients in stage III-IV (FIGO) had the highest levels of Ang-2, which can suggest the key role of Ang-2 in the development of tumor and metastases. Moreover, much higher serum Ang-2 levels in patients with LVSI might also reveal the fact that Ang-2 is a potential biomarker that can be used in the evaluation of staging. Although some evidence suggests that the expression of Ang-2 in tumor tissue indicates poor prognosis, only a few studies evaluated the levels of Ang-2 in circulation [46–50]. However, so far there has been no uniform conclusion. According to our current data, the level of serum Ang-2 might be a key prognostic factor in type 1 EC. Our results suggest better prognosis in patients with lower serum Ang-2 levels before treatment.

Excess adipose tissue is associated with elevated levels of the pro-inflammatory CRP marker in the bloodstream. Elevated levels of CRP and its IL-6 inducer might also serve as a predictive factor of the development of type 1 EC [11]. In a large population-based case-control study, a statistically significant 1.25 higher risk of EC type I development per unit of CRP growth was observed. Not many clinical trials so far have investigated the relationship between inflammatory markers and the risk of EC development. Research from the European Investigation into Cancer cohort indicates statistically significant increased risks of EC for elevated levels of prediagnostic, prospectively controlled CRP levels (OR = 1.58, 95% CI: 1.03–2.41) [5, 11]. In the clinical study described by Gathirua-Mwangi et al. [51] focused on 10,014 women aged 18 years and older who participated in NHANES III (Third National Health and Nutrition Examination Survey) a total of 400 cases of cancer deaths were documented, with 140 cases of deaths from obesity-related cancers (breast, colorectal, pancreatic and endometrial). Cox proportional hazards regression was used to estimate multivariable-adjusted hazard ratios (HR) for cancer mortality. Metabolic syndrome and CRP were associated with increased total cancer mortality [HR = 1.33, 95% CI 1.04–1.70]. In our study, this value was HR = 1.37, 95% CI: 1.03–1.61 for CRP, respectively. Our results correspond to previous reports on CRP in EC, where elevated serum CRP levels were associated with more aggressive tumor behavior, higher tumor development stages, and poor prognosis. Multivariable analysis showed that CRP is an independent prognostic factor in patients with type 1 EC with significantly worse overall prognosis. Higher preoperative levels of CRP were related to increased mortality. Measurements of CRP in type 1 EC could be easily integrated into routine diagnostic procedures. However, it is known that CRP is not a specific tumor marker for EC, therefore exclusion of other reasons for increased serum CRP before suspecting EC is crucial. Only then, the information on CRP serum concentrations in type 1 EC could be evaluated in relation to tumor stage or prognosis and could be used to support decisions about adjuvant chemotherapy.

## Conclusions

In conclusion, our research has shown that obesity-related angiogenesis and inflammation are related to the development of type 1 EC and survival. In our study, a significant correlation was found between serum Ang-2 and CRP levels and outcome of the patients with type 1 EC. The obtained results suggest that the levels of Ang-2 and CRP in the blood could be used as prognostic factors both, in diagnosis and in the treatment of type 1 EC. Moreover, the correlation between these proteins and type 1 EC suggests that it might potentially serve as a marker helping to predict the prognosis and to offer the possibility of customizing the treatment regimen. It is believed that the combined measurement of currently used tumor markers will improve sensitivity and specificity for type 1 EC management. However, the values of longitudinal measurements of the used markers before and after therapy have not yet been determined.

## Data Availability

The datasets generated during and/or analysed during the current study are not publicly available as study participants were assured raw data would remain confidential and not be shared.

## Abbreviations

type 1 EC: Endometrioid endometrial cancer
CRP: C-reactive protein
VEGF-A: Vascular endothelial growth factor-A
Ang-2: Angiopoietin-2
IGF-1: Insulin-like growth factor-1
LVSI: Lymphatic vascular space invasion
FIGO: International Federation of Gynecology and Obstetrics
TNF-α: Tumor necrosis factor α
IL-6: Interleukin-6
IGFBP2: Insulin like growth factor binding protein 2
SHBG: Sex hormone binding globulin
HGF: Hepatocyte growth factor
NHANES III: Third National Health and Nutrition Examination Survey
